# Keratoelastoidosis marginalis as a marker of cumulative actinic damage: A scientific letter

**DOI:** 10.4102/safp.v68i1.6397

**Published:** 2026-09-22

**Authors:** Kirstin Becker, Vanessa Kriek, Kevin McCree, Willem I. Visser

**Affiliations:** 1 Independent Researcher, Worcester, South Africa; 2 Division of Dermatology, Department of Medicine, Faculty of Medicine and Health Sciences, Tygerberg Academic Hospital, Cape Town, South Africa; 3 Division of Dermatology, Department of Medicine, Faculty of Medicine and Health Sciences, Stellenbosch University, Cape Town, South Africa; 4 Division of Anatomical Pathology, Faculty of Medicine and Health Sciences, Tygerberg Academic Hospital, Cape Town, South Africa; 5 Division of Anatomical Pathology, Faculty of Medicine and Health Sciences, Stellenbosch University, Cape Town, South Africa

**Keywords:** keratoelastoidosis marginalis, actinic damage, skin cancer risk, non-melanoma skin cancer, primary care dermatology, preventive care, benign skin lesions, skin cancer surveillance

## Abstract

**Background:**

Keratoelastoidosis marginalis (KEM) is a rare, acquired marginal keratoderma typically affecting older individuals with long-term sun exposure and manual labour. Although regarded as benign, its broader clinical relevance is often overlooked.

**Methods:**

We report a single case of histologically confirmed KEM identified during routine skin cancer surveillance in a high-risk patient. Clinical findings were correlated with histopathology and associated cutaneous diagnoses.

**Results:**

A 65-year-old fair-skinned male farmer with extensive occupational sun exposure presented with characteristic linear keratotic plaques along the margins of both hands. Histology demonstrated prominent solar elastosis with degenerated collagen and elastic fibres, consistent with KEM. During the same consultation, the patient was diagnosed with a synchronous basal cell carcinoma. No malignant transformation was identified within the KEM lesions themselves.

**Conclusion:**

This case highlights KEM as a visible marker of cumulative actinic damage rather than an isolated keratoderma.

**Contribution:**

Recognition of KEM in primary care and dermatology settings may assist clinicians in identifying patients at increased risk of keratinocyte skin cancer and prompt comprehensive skin examination, preventive counselling and appropriate surveillance.

## Introduction

Keratoelastoidosis marginalis (KEM) is an uncommon, acquired disorder of keratinisation classified within the spectrum of marginal papular keratodermas.^1,2,3^ It presents as asymptomatic, linear, waxy translucent papules and plaques along the junction of palmar and dorsal skin, most often affecting the radial aspect of the index finger and the ulnar aspect of the thumb.^1,3,4,5,6,7^ Keratoelastoidosis marginalis has been consistently associated with chronic ultraviolet (UV) exposure and repetitive manual trauma, predominantly in fair-skinned, middle-aged to elderly individuals.^1,2,3,5,6,7^

Despite its benign nature, KEM frequently occurs in patients with extensive actinic damage.^1,2,3,4,5,6,7^ Keratoelastoidosis marginalis is a rare condition whose potential role as a clinical marker of cumulative UV exposure and increased skin cancer risk may not be widely recognised in the primary care setting. In South Africa and other low- and middle-income countries (LMICs), where limited access to dermatology services places primary care clinicians at the forefront of diagnosing and managing skin disease, greater awareness of KEM may facilitate earlier recognition and appropriate screening for cutaneous malignancies in high-risk patients with cumulative actinic damage.^8^ This scientific letter presents a case of KEM identified during routine skin cancer surveillance and discusses its relevance for clinicians involved in long-term care of patients with chronic sun exposure.

## Case presentation

A 65-year-old male farmer was reviewed at a tertiary-level Dermatology clinic as part of routine follow-up for actinic keratoses and non-melanoma skin cancer. He reported lifelong occupational sun exposure with minimal use of protective clothing or gloves. There was no personal or family history of inherited keratodermas, and no exposure to chemical irritants or allergens.

Clinical examination revealed Fitzpatrick skin type II with marked signs of chronic photodamage. Both hands showed symmetrical, linear arrays of firm, angular to oval keratotic papules with occasional central depressions, coalescing into waxy plaques along the radial margins of the index fingers and ulnar margins of the thumbs, extending into the first web spaces (Figure 1). The lesions were asymptomatic.

**FIGURE 1:**
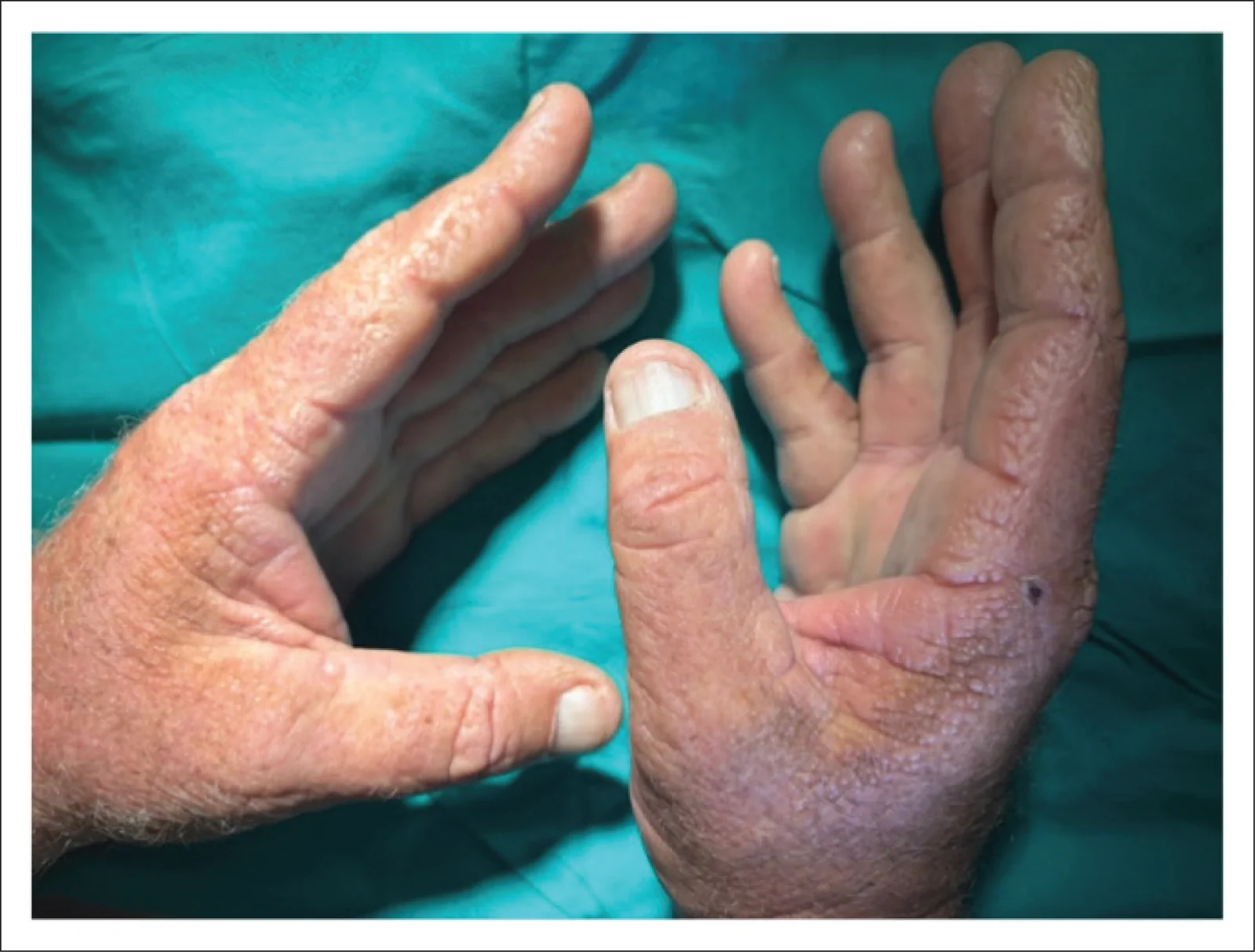
Clinical photograph taken of the patient’s hands showing symmetrical, linear keratotic papules and waxy plaques along the radial margins of the index fingers and ulnar margins of the thumbs, extending into the first web spaces, consistent with keratoelastoidosis marginalis. The surrounding skin demonstrates features of chronic actinic damage. Photograph taken by Vanessa Kriek, 2025 at Tygerberg Hospital, Cape Town.

A punch biopsy from a representative lesion revealed mild epidermal acanthosis with compact orthokeratosis. The underlying dermis demonstrated prominent solar elastosis, characterised by basophilic, thickened and fragmented elastic fibres with associated disorganisation, together with thickened, haphazardly arranged collagen bundles. No epidermal dysplasia or invasive malignancy was identified. These clinicopathological findings were consistent with a diagnosis of keratoelastoidosis marginalis.

During the same consultation, additional lesions were biopsied, one of which confirmed a basal cell carcinoma on the nose. The patient was counselled regarding the benign nature of KEM, the lack of effective lesion-directed therapy, and the importance of ongoing photoprotection and regular skin cancer surveillance.

## Discussion

Keratoelastoidosis marginalis is most frequently described in individuals with prolonged sun exposure and a history of manual labour.^1,2,3,5,6,7^ Histologically, the condition is characterised by marked solar elastosis and degeneration of dermal collagen and elastic fibres, reflecting chronic UV-induced dermal damage.^2,3,4,6,7^ These clinicopathological features suggest that KEM represents a manifestation of cumulative actinic injury rather than a purely mechanical keratoderma.

In the present case, the coexistence of KEM with extensive photodamage and a synchronous basal cell carcinoma is clinically relevant. A direct causal relationship between KEM and keratinocyte malignancy has not been established; however, the presence of KEM may serve as a readily visible cutaneous marker of long-standing carcinogenic UV exposure. Although KEM in this patient was identified during established skin cancer surveillance, rather than prompting the initial evaluation, the coexistence of KEM and skin malignancy reinforces its potential value as a clinical marker of cumulative ultraviolet damage. This case therefore highlights the importance of recognising KEM in primary care, where its identification may prompt a more thorough skin examination and appropriate skin cancer surveillance. Although the incidence of skin cancer is lower than in high-income countries, mortality is disproportionately higher in LMICs owing to delayed diagnosis and treatment, driven by shortages of dermatologists, inadequate healthcare infrastructure, limited dermatology training, financial barriers to care and poor health-seeking behaviour.^9^ Consequently, improving dermatology education and diagnostic capacity among primary care practitioners has been identified as a key strategy to facilitate earlier detection of skin malignancies.^8,9^ From a primary care and family practice perspective, recognising KEM should prompt comprehensive skin examination, a low threshold for biopsy of suspicious lesions, and reinforcement of sun-protective behaviours.

The differential diagnosis of marginal hand keratoses includes actinic keratoses, squamous cell carcinoma in situ, viral warts and hereditary marginal keratodermas.^1,2,3,6^ Hereditary marginal keratodermas typically present earlier in life and are often associated with a family history, in contrast to KEM, which is an acquired condition occurring later in adulthood.^1,2,3,6^ In sun-damaged skin, these entities may coexist, and histological confirmation is useful when the diagnosis is uncertain or when lesions change in appearance.

Pigmented or blue-coloured keratoelastoidosis-like lesions have been described in association with alkaptonuria and represent a distinct clinicopathological entity related to ochronosis rather than classical KEM.^10^

There is currently no consistently effective treatment for KEM. Reported therapeutic options have included topical keratolytics (such as urea or salicylic acid), topical retinoids, high-potency topical corticosteroids, cryotherapy and laser therapy, with variable and often disappointing results.^1,2,3,7^ Lesions are typically asymptomatic and chronically progressive, and treatment is therefore largely conservative.^3,4,7^

From a practical clinical perspective, management should focus on patient education, reassurance regarding the benign nature of the condition, and preventive strategies aimed at limiting further actinic damage.^1,2,7^ Regular use of broad-spectrum sunscreen, protective clothing and gloves during outdoor work may help reduce progression.^1,2,7^ Given the frequent coexistence of KEM with significant photodamage, ongoing skin cancer surveillance remains a key component of management.

## Conclusion

Keratoelastoidosis marginalis is a benign condition but may represent an important clinical marker of cumulative actinic damage. Its recognition in patients with significant sun exposure should alert clinicians to an increased risk of keratinocyte skin cancer and prompt appropriate screening, prevention and follow-up strategies.

## References

[1] Coutada A, Butina MR, Luzar B. Keratoelastoidosis marginalis of the hands: A distinct form of palmoplantar keratoderma. Cureus. 2024;16(11):e73515. 10.7759/cureus.73515 PMC11636050 39669842

[2] Sharma S, Aggarwal K, Kumar S, Dalal N, Pathi RL, Sangal B. Keratoelastoidosis marginalis of the hands: Could it also involve the feet? Photodermatol Photoimmunol Photomed. 2021;37(1):71–73. 10.1111/phpp.12591 32652748

[3] Bhobe M, Tambe S, Jerajani H, Parulkar P. Keratoelastoidosis marginalis of the hands: A report in two farmers. Indian Dermatol Online J. 2016;7(3):195–197. 10.4103/2229-5178.182364 PMC4886595 27294058

[4] Burks JW, Montgomery H, Henderson F. Degenerative collagenous plaques of the hands. Arch Dermatol. 1960;82:454–458. 10.1001/archderm.1960.01580030056007 13849242

[5] Kocsard E. Keratoelastoidosis of the hands. Dermatologica. 1964;131:169–175. 10.1159/000254469 4222410

[6] Rahbari H. Acrokeratoelastoidosis and keratoelastoidosis marginalis: Any relation? J Am Acad Dermatol. 1981;5(3):348–350. 10.1016/S0190-9622(81)80159-4 7263981

[7] Mengesha YM, Kayat JD, Swerlick RA. Keratoelastoidosis marginalis. J Cutan Med Surg. 2002;6(1):23–25. 10.1007/s10227-001-0025-x 11896420

[8] Makaula PU, Chateau AV, Hift RJ, Dlova NC, Mosam A. The impact of basic dermatology education and training on primary healthcare providers in KwaZulu-Natal, South Africa. S Afr Fam Pract. 2021;63(1):a5200. 10.4102/safp.v63i1.5200 PMC8378187 33567838

[9] Maatouk C, Lucero-Prisno DE III. Barriers to skin cancer diagnosis and treatment in low- and middle-income countries and solutions: A literature review. Public Health Chall. 2025;4(1):e70042. 10.1002/puh2.70042 PMC12039339 40496102

[10] Sharma A, Mustari AP, Singh S, Chatterjee D, Kumaran MS. Blue-coloured keratoelastoidosis marginalis-like lesions in two middle-aged women. Clin Exp Dermatol. 2024;49(4):426–429. 10.1093/ced/llad421 38039143

